# Crystal structure of bacterial ubiquitin ADP-ribosyltransferase CteC reveals a substrate-recruiting insertion

**DOI:** 10.1016/j.jbc.2023.105604

**Published:** 2023-12-28

**Authors:** Zhengrui Zhang, Hannah M. Rondon-Cordero, Chittaranjan Das

**Affiliations:** Department of Chemistry, Purdue University, West Lafayette, Indiana, USA

**Keywords:** ADP-ribosylation, bacterial toxin, ubiquitin, protein crystallization, isothermal titration calorimetry, host–pathogen interaction

## Abstract

ADP-ribosylation is a post-translational modification involved in regulation of diverse cellular pathways. Interestingly, many pathogens have been identified to utilize ADP-ribosylation as a way for host manipulation. A recent study found that CteC, an effector from the bacterial pathogen *Chromobacterium violaceum*, hinders host ubiquitin (Ub) signaling pathways *via* installing mono-ADP-ribosylation on threonine 66 of Ub. However, the molecular basis of substrate recognition by CteC is not well understood. In this article, we probed the substrate specificity of this effector at protein and residue levels. We also determined the crystal structure of CteC in complex with NAD^+^, which revealed a canonical mono-ADP-ribosyltransferase fold with an additional insertion domain. The AlphaFold-predicted model differed significantly from the experimentally determined structure, even in regions not used in crystal packing. Biochemical and biophysical studies indicated unique features of the NAD^+^ binding pocket, while showing selectivity distinction between Ub and structurally close Ub-like modifiers and the role of the insertion domain in substrate recognition. Together, this study provides insights into the enzymatic specificities and the key structural features of a novel bacterial ADP-ribosyltransferase involved in host–pathogen interaction.

ADP-ribosylation is a reversible post-translational modification (PTM), in which the ADP ribose (ADPR) moiety from NAD^+^ is transferred onto substrates with the release of nicotinamide (NAM) (1). One of the major enzyme families catalyzing ADP-ribosylation is the group of ADP-ribosyltransferases (ARTs). Based on the active site residues, ARTs can be further classified into two subclasses: the diphtheria toxin–like ARTs and the cholera toxin–like ARTs (ARTCs) families (1, 2, 3, 4, 5). The diphtheria toxin–like ARTs often harbor either an H-Y-[EDQ] or H-H-Φ (Φ denotes a hydrophobic/aromatic residue) sequence motif in their active sites and catalyze mono-ADP-ribosylation (MARylation) or poly-ADP-ribosylation, whereas ARTCs harbor an R-[ST]-E motif and catalyze only MARylation (3). In humans, ADP-ribosylation is closely related to stress response (*e.g.*, DNA damage repair) (6, 7, 8) and other PTMs like ubiquitination (9, 10, 11, 12, 13). Interestingly, besides its roles in regulating normal cellular processes, ADP-ribosylation also serves as a means employed by pathogens to interfere with host signaling pathways. A considerable number of secreted bacterial proteins (effectors) have been identified to target host proteins *via* MARylation, including eukaryotic elongation factor 2 (14, 15, 16) and actin (17, 18, 19, 20, 21, 22). A striking case of bacteria-mediated ADP-ribosylation is exemplified by the SidE effector family in the pneumonia-causing pathogen, *Legionella pneumophila* (23, 24, 25). Specifically, the SidE effectors catalyze a two-step atypical ubiquitination on various host targets, starting with MARylation on ubiquitin (Ub) Arg42. This MARylated Ub is then added onto host targets by the phosphodiesterase domain in the same effector, forming a phosphoribosyl linker between Ub Arg42 and serine residues of the targets, with the concomitant release of AMP. Recently, another bacterial effector CteC, from *Chromobacterium violaceum*, has been found to specifically MARylate Ub but not Ub-like proteins (UBLs). The MARylation occurs on Thr66 of Ub, resulting in an ADP-ribosylated Ub derivative that is nonfunctional in a variety of Ub-related processes ultimately leading to the malfunction of host Ub system (26). Regarding enzyme classification, CteC belongs to the ARTC family with an R-S-E motif (R65, S97, and E220), with R65 and E220 being catalytically essential residues (26).

*C. violaceum* is an opportunistic Gram-negative bacterial pathogen that causes human skin lesions, sepsis, and liver abscesses (27). It harbors two type III secretion systems (T3SSs), Cpi-1/-1a and Cpi-2, with Cpi-1/-1a being critical for bacterial survival during infection (28). The Cpi-2 T3SS, on the other hand, does not seem to affect the infectivity or bacterial survival and is presumed to play a regulatory role during infection (28). The Ub-targeting mono-ART (mART) effector CteC is reported to be secreted through Cpi-1/-1a T3SS (29). This modification on Ub dampens several aspects of Ub system, including Ub transfer from the Ub-activating E1 enzyme to the Ub-conjugating E2, protein degradation by ubiquitin–proteasome system, and poly-Ub recognition by poly-Ub interactors (26). Aside from the biological evidence showing the importance of this effector during bacterial infection, the molecular basis of this Ub MARylation has been probed by a recent structural study (30). In this study, the authors captured CteC structures bound to Ub or ADPR-Ub, showing the CteC–Ub interface. They also captured interaction of CteC with the nucleotide donor although with missing electron density for adenosine (30).

Here, we provide biochemical evidence further strengthening the specificity of CteC toward Ub, but not other UBLs (NEDD8, SUMO1, and ISG15), for ADP-ribosylation. In addition, CteC displays residue-level selectivity for MARylating hydroxyl side chains, threonine and serine, but not tyrosine. The enzyme accepts, although less efficiently, the thiol group of cysteine as the nucleophile for MARylation but not arginine or asparagine. We also present a 1.87 Å crystal structure of CteC in complex with NAD^+^, which reveals a canonical mART fold while featuring an additional insertion domain placed within the core mART domain. The NAD^+^ binding pocket in CteC is well defined by electron density showing the full NAD^+^ molecule. Subsequent biochemical studies validate the key interactions observed in the NAD^+^ binding site and show that the insertion domain is indispensable for Ub interaction. Together, this study provides insights into the enzymatic specificities and the key structural features of CteC-mediated Ub ADP-ribosylation.

## Results

### Substrate and residue-level specificity of CteC

**Figure 1:**
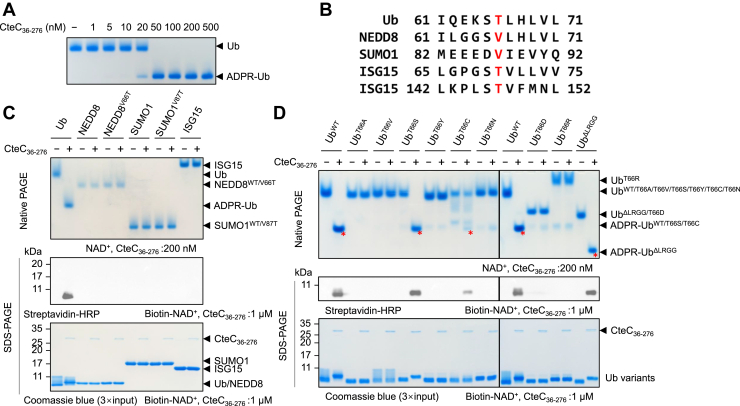
**Specificity of CteC-mediated ubiquitin (Ub) mono-ADP-ribosylation (MARylation).***A*, concentration-dependent Ub MARylation by CteC. *B*, sequence comparison of Ub to UBLs. Residues corresponding to Ub T66 are marked *red*. *C*, protein substrate specificity of CteC. *D*, residue specificity of CteC. For (*C*) and (*D*), reactions using NAD^+^ were analyzed *via* native PAGE and stained by Coomassie *blue*. Reactions using biotin–NAD^+^ were analyzed by Western blotting following SDS-PAGE, with loading shown *via* SDS-PAGE and stained by Coomassie *Blue*. In (*D*), ADPR-Ub on native PAGE was marked by *red asterisks*. ADPR, ADP ribose; UBL, Ub-like protein.

In our attempts to recombinantly express and purify CteC, we found that the full-length construct did not express and purify well. We therefore analyzed the CteC sequence using PlaToLoCo web server (31), which indicated that N-terminal residues 1 to 35 may be disordered. We therefore cloned, expressed, and purified the CteC_36–276_ construct. This construct was well behaved in purification and was thus used for our subsequent biochemical and structural studies. To test if CteC_36–276_ exhibits normal mART activity as the full-length construct, we performed the Ub ADP-ribosylation assay with different concentrations of CteC. The formation of ADPR-Ub was monitored using a higher electrophoretic mobility in native PAGE because of the extra negative charge of the ADPR group, which showed that even as low as 50 nM CteC can readily convert almost all 100 μM Ub into ADPR-Ub within 10 min at room temperature (Fig. 1*A*).

To further probe the substrate specificity of CteC, different UBLs, including NEDD8, SUMO1, and ISG15, were tested as potential substrates for CteC. Structurally, these UBLs share the same β-grasp fold of Ub. Sequence alignment of UBLs with Ub suggested that the residues corresponding to Ub T66 are conserved in both Ub-like domains in ISG15, whereas valine is in place of threonine at this position in NEDD8 and SUMO1 (Fig. 1*B*). Therefore, NEDD8^V66T^ and SUMO1^V87T^ were also included as potential substrates. We probed the MARylated product using the aforementioned native PAGE mobility assay and anti-biotin immunoblotting with biotin-NAD^+^ as the substrate. Interestingly, despite the structural similarities toward Ub, none of the UBLs was accepted by CteC for ADP-ribosylation (Fig. 1*C*), indicating that the substrate recognition by CteC is highly specific and beyond just the threonine residue. This is particularly striking for NEDD8, which shares nearly 58% sequence identity with Ub. Placement of Thr at the correct location in this UBL does not confer enzymatic modification despite high structural similarity, indicating that CteC has evolved to subtly distinguish Ub from its close cousin. Next, we tested residue-level selectivity of CteC by asking if the ADP-ribosylation can only be installed on threonine. Toward this goal, a panel of Ub T66 mutants was generated and tested for ADP-ribosylation by CteC. The results showed that only WT or T66S Ub can be robustly ADP-ribosylated by CteC, whereas T66C mutant also showed detectable ADP-ribosylation (Fig. 1*C*). Since other mutations at this position cannot be ADP-ribosylated by CteC, it appears that a small side chain on Ub is required for CteC recognition, with the hydroxyl group (threonine or serine) preferred over the thiol group of cysteine. Inability to accept tyrosine could imply spatial restriction imposed by active site pocket that favors smaller side chains. In addition, removing C-terminal LRGG residues from Ub does not affect the MARylation by CteC (Fig. 1*D*), showing that the C terminus of Ub is likely not recognized by CteC, implying that Ub can be modified with tethered C terminus, as in cases of ubiquitinated proteins or poly-Ub chains.

### Crystal structure of CteC

To understand the structural basis of the ADP-ribosylation by CteC, we first examined the CteC structure predicted by AlphaFold (32, 33). Surprisingly, the overall predicted structure of CteC is of low confidence, and a considerable proportion was predicted to be unstructured (Fig. S1*A*). Not surprisingly, this model was unable to provide phase estimation in molecular replacement searches with diffraction datasets of native CteC. Therefore, we expressed and purified the selenomethionine-substituted CteC_36–276_ (SeMet-CteC_36–276_), crystallized it in complex with NAD^+^, and determined its structure at 1.87 Å *via* single-wavelength anomalous diffraction (SAD) phasing (Table S1). The NAD^+^-bound SeMet-CteC_36–276_ crystallized in *P*2_1_ 2_1_ 2_1_ space group, with three copies in one asymmetric unit.

**Figure 2:**
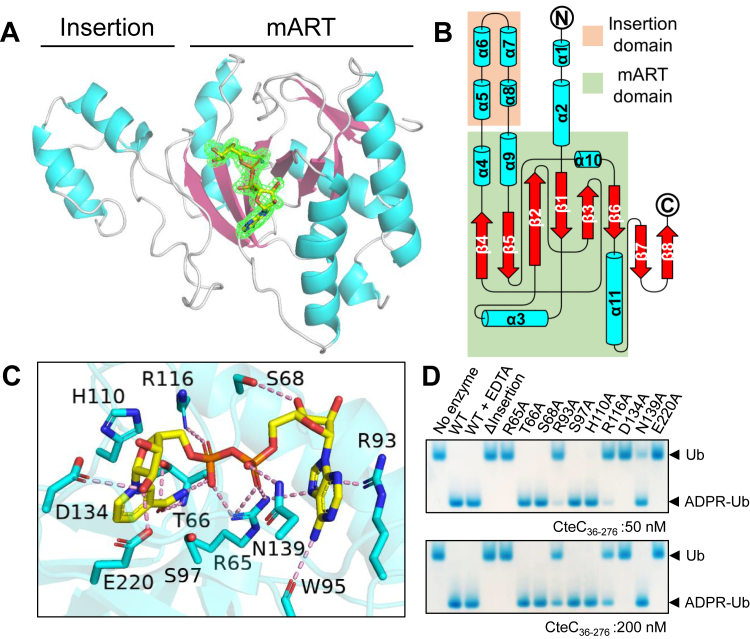
**Structure of NAD**^**+**^**-bound CteC**_**36–276**_**.***A*, overall structure of NAD^+^-bound CteC_36–276_. mART domain and insertion domain are marked, with the electron density of NAD^+^ shown by *F*_o_–*F*_c_ map (contour = 3σ). *B*, topology diagram of CteC_36–276_. *C*, interactions between NAD^+^ and CteC. *D*, activity assay probing crucial structural features affecting CteC activity. All reactions were performed using 50 nM and 200 nM CteC_36__–__276_, analyzed *via* native PAGE, and stained by Coomassie *Blue*. mART, mono-ART.

The CteC structure harbors two domains, a core mART domain with NAD^+^ bound and a small helical insertion domain with no apparent interaction with NAD^+^ (Fig. 2*A*). The mART domain in CteC follows a canonical mART fold (3), in which a six-stranded β-sheet is observed following an order of β4-β5-β2-β1-β3-β6 (Fig. 2*B*). This β-sheet can be further dissected into two subunits of interlaced β-strands, consisting of β4-β5-β2 and β2-β1-β3-β6, with β2 being a curved strand involved in both units. The insertion domain in CteC is a small four-helical bundle present between β4 and β5, spanning from residue F157 to R194 (Fig. S1*C*). Interestingly, we found that the AlphaFold-predicted CteC structure has a high confidence in this insertion domain, with a Cα RMSD of 0.391 Å compared with our structure (Fig. S1*D*). DALI search using this insertion domain did not reveal any Ub-related or ADP-ribosylation-related hits. On the other hand, the R-S-E motif in CteC follows a sequential residue arrangement similar in other ARTCs, including Iota toxin (17), C3 toxin (22), and TccC3 toxin (21) (Fig. S1*C*), indicating that CteC adopts basic features of the ARTC family.

### NAD^+^ binding in CteC

Within the mART domain, every atom of NAD^+^ can be unambiguously placed in its binding pocket based on the electron density (Fig. 2*A*) in all three copies in the asymmetric unit. Overall, NAD^+^ binds CteC in a bent conformation *via* multiple interactions (Fig. 2*C*). Specifically, the adenine base is primarily positioned by the π–cation interaction with the guanidinium group of the R93 side chain, with additional interactions with W95 carbonyl oxygen and R65 side chain *via* N6 and N7 atoms, respectively. The C3′ hydroxyl group in the adenosine ribose forms one hydrogen bonding with the S68 side chain. The diphosphate group is held by the R65 side chain, with additional polar interactions of α-phosphate to N139 side chain and β-phosphate to R113 side chain. The NAM ribose is held *via* hydrogen bonding between C2’’ hydroxyl group and the side chains of D134 and E220. The NAM group is positioned by backbone nitrogen, oxygen, and side chain hydroxyl group of T66. Within the R-S-E motif, R65 interacts with both adenine base and the diphosphate group, whereas E220 interacts with the NAM ribose. S97 in the R-S-E motif, despite being in close proximity to the NAD^+^ binding pocket, does not form apparent interaction with NAD^+^ (Fig. 2*C*). Curiously, we observed a Ca^2+^ ion near the NAM ribose, coordinating with C2’’ and C3’’ hydroxyl groups, D134 side chain, A135 backbone carbonyl oxygen, and three water molecules (Fig. S2).

### Structural features affecting CteC activity

Based on our structure, we sought to explore important substrates and residues that are vital for CteC activity. First, to investigate if this insertion domain is directly related to Ub MARylation, we designed a CteC construct with the insertion domain removed (referred to as CteC_36–276_^ΔINS^). Specifically, in this construct, residues K158 and S195 were directly linked *via* a -Gly-Ser-linker (Fig. S3*A*). We performed CD spectroscopy of CteC_36–276_ and CteC_36–276_^ΔINS^ to confirm the folding behavior of the protein upon insertion domain removal. The normalized CD spectrum showed that both CteC_36–276_ and CteC_36–276_^ΔINS^ are well folded, yet CteC_36–276_^ΔINS^ has less α-helical structures (Fig. S3*B*), which can be attributed to the loss of helical content resulting from the absence of the four helices of the insertion domain. However, our biochemical assay showed no detectable MARylation catalyzed by CteC_36–276_^ΔINS^ (Fig. 2*D*), indicating that the insertion domain is required for CteC activity even though it is not required for the folding of the mART domain.

Next, we generated a panel of CteC mutants based on the observed NAD^+^ interactions and tested their activity (Fig. 2*D*). We found that R93A, R113A, and N139A mutants exhibited noticeably dampened activities, indicating that both adenine and the diphosphate need to be properly placed for optimal CteC activity. This is also supported by the observation that the R65A mutant showed no activity in our assay. In addition, the D134A and E220A mutants also lost their MARylation activity, suggesting that both residues play critical catalytic roles in CteC. In the R-S-E motif, the glutamate (E220 in CteC) is believed to facilitate the release of NAM and to stabilize the resulting oxocarbenium ion (1, 3). We speculate that D134 could play similar roles, with an additional role in possibly helping to extract the hydroxyl proton of Ub T66, activating the threonine hydroxyl group for nucleophilic attack. With regard to the observation of Ca^2+^ near active site in our crystal structure, inclusion of EDTA in reaction mixture does not affect CteC activity (Fig. 2*D*), indicating that the Ca^2+^ found near NAD^+^ binding site does not participate in catalysis, meaning that CteC is not a metalloenzyme. As our crystallization condition includes calcium acetate (see the Experimental procedures), we reason that the Ca^2+^ observed in our structure likely comes from the crystallization buffer. It might have helped stabilize the observed conformation of certain residues in the vicinity of NAD^+^ binding pocket in the crystals of CteC.

### CteC insertion domain is required for Ub binding

**Figure 3:**
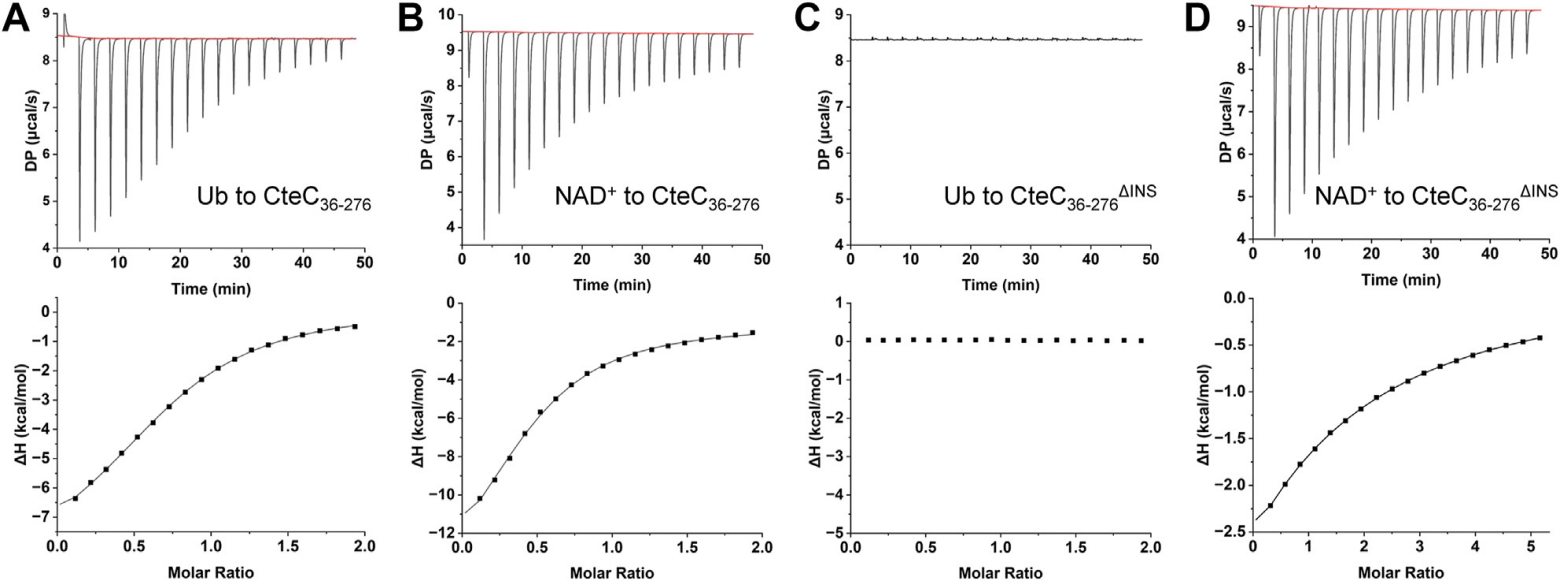
**Isothermal titration calorimetry profile.***A* ubiquitin (Ub) to CteC_36–276_, (*B*) NAD^+^ to CteC_36–276_, (*C*) Ub to CteC_36–276_^ΔINS^, and (*D*) NAD^+^ to CteC_36–276_^ΔINS^.

As the insertion domain in CteC is needed for activity, but does not interact directly with NAD^+^, we asked whether CteC_36–276_^ΔINS^ exhibits altered affinity for Ub, which may explain the loss of activity when the insertion is deleted from the protein. To this end, we compared the binding affinity of CteC_36–276_ and CteC_36–276_^ΔINS^ for Ub and NAD^+^ measured using isothermal titration calorimetry (ITC). The equilibrium dissociation constants (*K*_*d*_) of CteC_36–276_ to Ub and NAD^+^ were very similar, 119 μM (Fig. 3*A*) and 123 μM (Fig. 3*B*), consistent with the reported values of 78.7 μM and 86.2 μM (26). However, CteC_36–276_^ΔINS^ did not show observable binding to Ub (Fig. 3*C*), indicating that the insertion domain plays an important role in Ub recognition. Interestingly, although our titration shows that CteC_36–276_^ΔINS^ still retains NAD^+^ binding (Fig. 3*D*), we cannot obtain a reliable *K*_*d*_ value because of the weak binding between these two molecules. This attenuated binding to NAD^+^ could be attributed to potential structural rearrangement of CteC near the NAD^+^ binding site upon the insertion domain removal. Thus, the insertion domain is primarily used for Ub recruitment although it may play a secondary role in NAD^+^ binding, even though the crystal structure shows the nucleotide binding pocket is spatially distinct from the position of the insertion.

## Discussion

Here, we present biochemical and structural characterization of CteC, a bacterial effector that MARylates Ub on threonine 66. This ADP-ribosylation interferes with the normal function of Ub in the E1–E2–E3-mediated Ub transfer in host cells. Such an impairment of ubiquitination, among other things, helps dampen innate immune response by blocking NF-кB activation (26). Prior to the discovery of the CteC, the only other example of threonine ADP-ribosylation was the actin ADP-ribosylation catalyzed by bacterial toxin TccC3 (21). Despite being structurally characterized in its apo and actin-bound forms, TccC3 was not captured in its NAD^+^-bound form experimentally (34). Nevertheless, together, these two examples have expanded our current knowledge on threonine ADP-ribosylation, which is previously unprecedented in the eukaryotic world. In addition to expanding the scope of ADP-ribosylation, these examples underscore the importance of this PTM in host–pathogen interaction. Therefore, the structure we report here could open new avenues for development of novel antimicrobials.

It is interesting that the AlphaFold-predicted structure of CteC does not provide enough structural insights, despite CteC being a relatively small protein. This could be attributed to the absence of previously determined structures of CteC homologs. In other words, the amino acid sequence of CteC may not be well represented structurally in current databases. We expect that the X-ray structure determination of CteC may help future structure prediction of CteC homologs.

CteC adopts a canonical mART fold with an additional insertion domain (Fig. 2*A*), which is needed for Ub binding (Fig. 3*C*) and catalytic activity (Fig. 2*D*). These results suggest that the insertion domain plays an essential role in Ub recruitment, which is also confirmed by the recent study showing the co-crystal structure of Ub-bound CteC, in which the CteC–Ub interface is observed between Ub and the insertion domain, with no involvement of the mART domain (30). In addition, the authors also reported a CteC mutant with double mutations in the insertion (M160E/F172E), which lost Ub binding (30). Interestingly, the catalytic domains of human poly(ADP-ribose) polymerases (PARPs) −1, −2, and −3 harbor helical domain—ART architectures similar to CteC. Yet, the helical domains in these PARPs are autoinhibitory modules that block productive NAD^+^ binding of the ARTs, which can be alleviated by PARP-activating signals (*e.g.*, binding to damaged DNA) (35). The structural characterization of CteC essentially provides an opposite example where an auxiliary domain helps substrate recruitment for ADP-ribosylation. It is tempting to speculate that in the evolutionary history, CteC might have started out with the mART domain and acquired the insertion as a later development as the bacterium needed to respond to the host defense. Although the evolutionary origin of the Ub-recruiting insertion remains unclear, our study adds to an emerging notion of modular construction of bacterial enzymes meant for host interaction: a blending of ancient enzymatic folds with newer substrate-recruiting modules (36).

Intriguingly, in the recently published Ub-bound CteC structure, the threonine side chain of Ub is quite a distance away from the NAD^+^ binding pocket (13.4 Å between the threonine side chain oxygen and the C1’’ of NAM ribose) (30), implying the role of protein dynamics in catalysis. It is possible that Ub first interacts with the insertion domain, inducing relative motion of mART and insertion domains, which brings the threonine residue into the active site. However, CteC only accepts residues with small side chains (Fig. 1*D*), suggesting that local conformation around the NAD^+^ binding pocket likely also helps Ub recognition. On the other hand, our crystal structure of NAD^+^-bound CteC reveals the full NAD^+^ binding mode, which will likely help further characterization on the mechanistic details of threonine MARylation. The bent conformation of NAD^+^ observed in our structure suggests a similar strain-alleviation model for ADPR transfer (1, 3). In this model, the strain induced in the bound nucleotide promotes an S_N_1 displacement reaction in which NAM is liberated to yield an oxocarbenium ion intermediate prior to the nucleophilic attack by the hydroxyl group. That mechanism will be consistent with serine also working robustly in the reaction under the conditions we have used in our assay. However, further structural investigations on CteC are required to confirm this proposed mechanism as well as the CteC^mART^–Ub interface in the catalytically productive orientation.

## Experimental procedures

### Cloning, plasmids, and mutagenesis

DNA fragments of CteC_36–276_, NEDD8_1–76_, SUMO1_1–97_, ISG15_1–156_^C78S^ were synthesized and cloned in pGEX6P1 using BamHI–XhoI restriction sites. DNA fragment of Ub was PCR-amplified and cloned in pET-DUET-1 using NdeI–XhoI restriction sites. Site-directed mutagenesis was performed by PCR amplifying the plasmid harboring the construct using mutagenic primer pairs. The methylated template plasmids were removed by the addition of DpnI. All the plasmids were verified by Sanger sequencing before further use.

### Recombinant protein expression and purification

For recombinant protein expression, bacterial expression plasmids were transformed into *Escherichia coli* BL21(DE3) strain. For native protein expression, the transformed cells were grown in LB media at 37 °C to an absorbance of 0.6 to 0.8 at 600 nm. The protein expression was induced by addition of 0.4 mM IPTG at 18 °C for 16 h. For SeMet-CteC_36–276_ expression, the transformed cells were grown in M9 minimal media at 37 °C to an absorbance of 0.6 to 0.8 at 600 nm. An amino acid mixture including 100 mg of l-lysine, 100 mg of l-phenylalanine, 100 mg of l-threonine, 50 mg of l-valine, 50 mg of l-isoleucine, 50 mg of l-leucine, and 50 mg of l-selenomethionine was then added, as solid, to each liter culture. The culture was further incubated at 37 °C for 15 min before inducing the protein expression with 0.4 mM IPTG at 18 °C for 16 h.

After expression, cells were collected *via* centrifugation at 7000*g* for 7 min and resuspended in 1× PBS with 0.4 M KCl. The resuspension was passed twice through a French press under 1500 psi, and the cell debris was removed by ultracentrifugation at 100,000*g* at 4 °C for 1 h. The glutathione-*S*-transferase–tagged protein in the supernatant was purified on the glutathione resin following the manufacturer’s instruction. For Ub purification, cells were collected, resuspended in 50 mM sodium acetate (pH 4.5), and heated in 80 °C water bath for 30 min. After ultracentrifugation at 100,000*g* at 4 °C for 1 h, Ub in the supernatant was captured using SP Sepharose (Cytiva) resin and eluted with 50 mM sodium acetate (pH 4.5) supplemented with 0.3 M NaCl. All the proteins were further purified by size-exclusion chromatography and stored in 50 mM Tris–HCl (pH 7.4), 50 mM NaCl, and 1 mM DTT (5 mM DTT for SeMet-CteC_36–276_). The purity of the protein was monitored by SDS-PAGE.

### Crystallization and data processing

To generate NAD^+^-bound CteC complex, NAD^+^ was dissolved in 50 mM Tris–HCl (pH 7.4), 50 mM NaCl, and 5 mM DTT, and added to SeMet-CteC_36–276_ with a final protein concentration of 20 mg/ml and a final NAD^+^ concentration of 5 mM. The mixture was incubated on ice overnight to allow complex formation before crystallization screening by hanging drop vapor diffusion method at 20 °C. After 24 h, several hits were observed. These conditions were replicated and optimized by altering salt and precipitant concentrations. Eventually, NAD^+^-bound SeMet-CteC_36–276_ crystals were obtained from the condition containing 0.25 M calcium acetate and 20% PEG3000 with 1:1 ratio of protein solution to reservoir solution. Crystals were observed after 3 h at 20 °C by hanging drop vapor diffusion in this condition. A complete dataset was collected from a single crystal at the Advanced Photon Source at Argonne National Laboratories on the GM-CA 23-ID-B beamline (λ = 0.9794 Å) using SAD. Initial data were processed and scaled using HKL3000 (HKL Research) (37) at 1.87 Å in *P*2_1_ 2_1_ 2_1_ space group. To determine the structure, AutoSol (38) in PHENIX suite (39) was used for SAD phasing. The initial structure went through multiple rounds of refinement using COOT (40) and PHENIX (39) to generate a final structure. The structure was validated by MolProbity (41) and deposited in the Protein Data Bank (code: 8UX2).

### ADP-ribosylation assays

To obtain the optimal CteC concentration for biochemical assays, CteC_36–276_ at the concentration ranging from 1 to 500 nM was incubated with 100 μM Ub in the buffer containing 25 mM Tris–HCl (pH 7.4), 75 mM NaCl, 1 mM DTT (assay buffer), with 1 mM NAD^+^ for 10 min at room temperature. Activities of CteC mutants were probed by incubating 50 nM or 200 nM CteC_36–276_ mutants with 100 μM Ub in assay buffer with 1 mM NAD^+^ for 10 min at room temperature. These reactions were analyzed on native PAGE and stained by Coomassie blue.

To test the ADP-ribosylation activity of CteC on Ub mutants, UBLs, and UBL mutants, 200 nM CteC_36–276_ was incubated with 50 μM Ub mutants or 20 μM UBL/UBL mutants in assay buffer with 1 mM NAD^+^ for 45 min at room temperature. These reactions were analyzed on native PAGE and stained by Coomassie blue. In addition, 1 μM CteC_36–276_ was incubated with 20 μM Ub mutants/UBL/UBL mutants, and 35 μM biotin-NAD^+^ (Biolog) in assay buffer for 90 min at room temperature. These reactions were quenched by 5× SDS loading dye and probed with biotinylation *via* Western blotting. Loading of these reactions was monitored by SDS-PAGE with Coomassie blue staining.

### CD spectroscopy

CteC_36–276_ and CteC_36–276_^ΔINS^ were buffer-exchanged in 10 mM potassium phosphate (pH 7.5) and diluted to 3 μM. The CD data were collected from 260 nm to 185 nm by JASCO J-1500 CD spectrophotometer using 1 mm cuvette. Data were normalized as molar ellipticity and plotted against wavelength.

### ITC

The ITC experiments were performed using MicroCal PEAQ-ITC (Malvern Panalytical). Specifically, for CteC_36–276_, 480 μM CteC_36–276_ was titrated with 4.8 mM Ub or NAD^+^ in the buffer containing 50 mM Hepes, pH 7.3, and 100 mM NaCl (ITC buffer). For CteC_36–276_^ΔINS^, 480 μM CteC_36–276_^ΔINS^ was titrated with 4.8 mM Ub in ITC buffer, and 750 μM CteC_36–276_^ΔINS^ was titrated with 20 mM NAD^+^ in ITC buffer. The raw data were integrated and analyzed by MicroCal PEAQ-ITC Analysis Software, version 1.41 (Malvern Panalytical) to determine the *K*_*d*_. Processed data were replotted using Origin 2023b (OriginLab) for presentation.

## Data availability

Structural factors and atomic coordinates of NAD^+^-bound SeMet-CteC_36–276_ have been deposited to Protein Data Bank with the accession code 8UX2.

## Supporting information

This article contains supporting information.

## Conflict of interest

The authors declare that they have no conflicts of interest with the contents of this article.

## Supporting information

Figure S3

Figures and Table Captions
