# Supplementary figures and images for: Crystal structure of bacterial ubiquitin ADP-ribosyltransferase CteC reveals a substrate-recruiting insertion
Source: J Biol Chem. 2023 Dec 28;300(2):105604. doi: 10.1016/j.jbc.2023.105604 (PMC10810742; doi:10.1016/j.jbc.2023.105604)

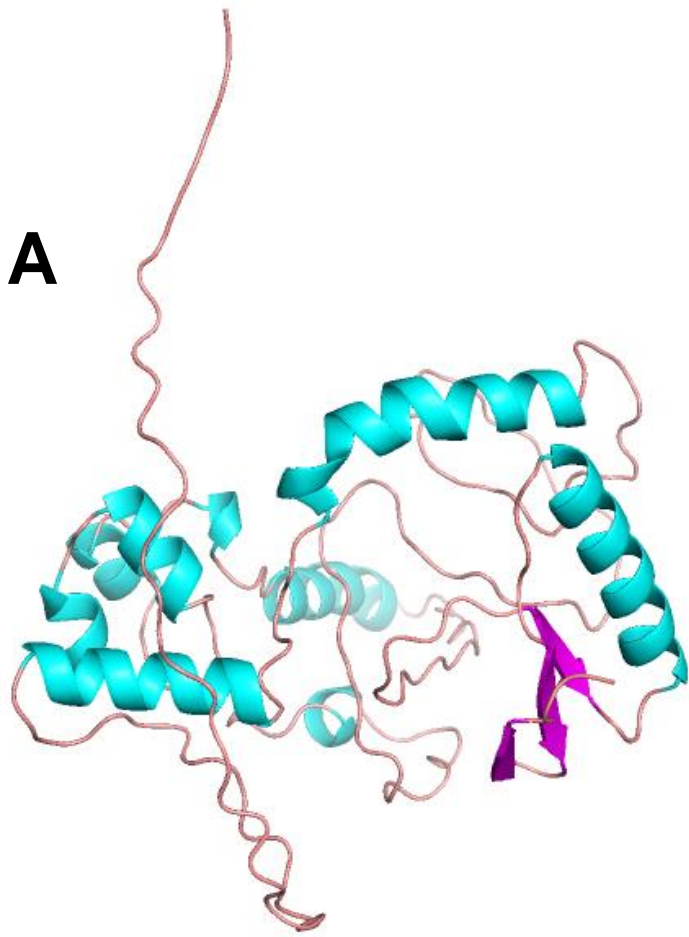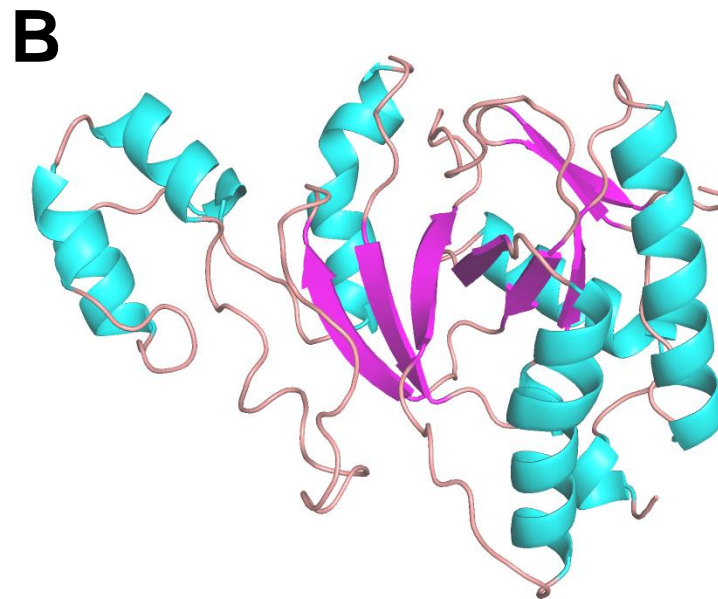

**C**

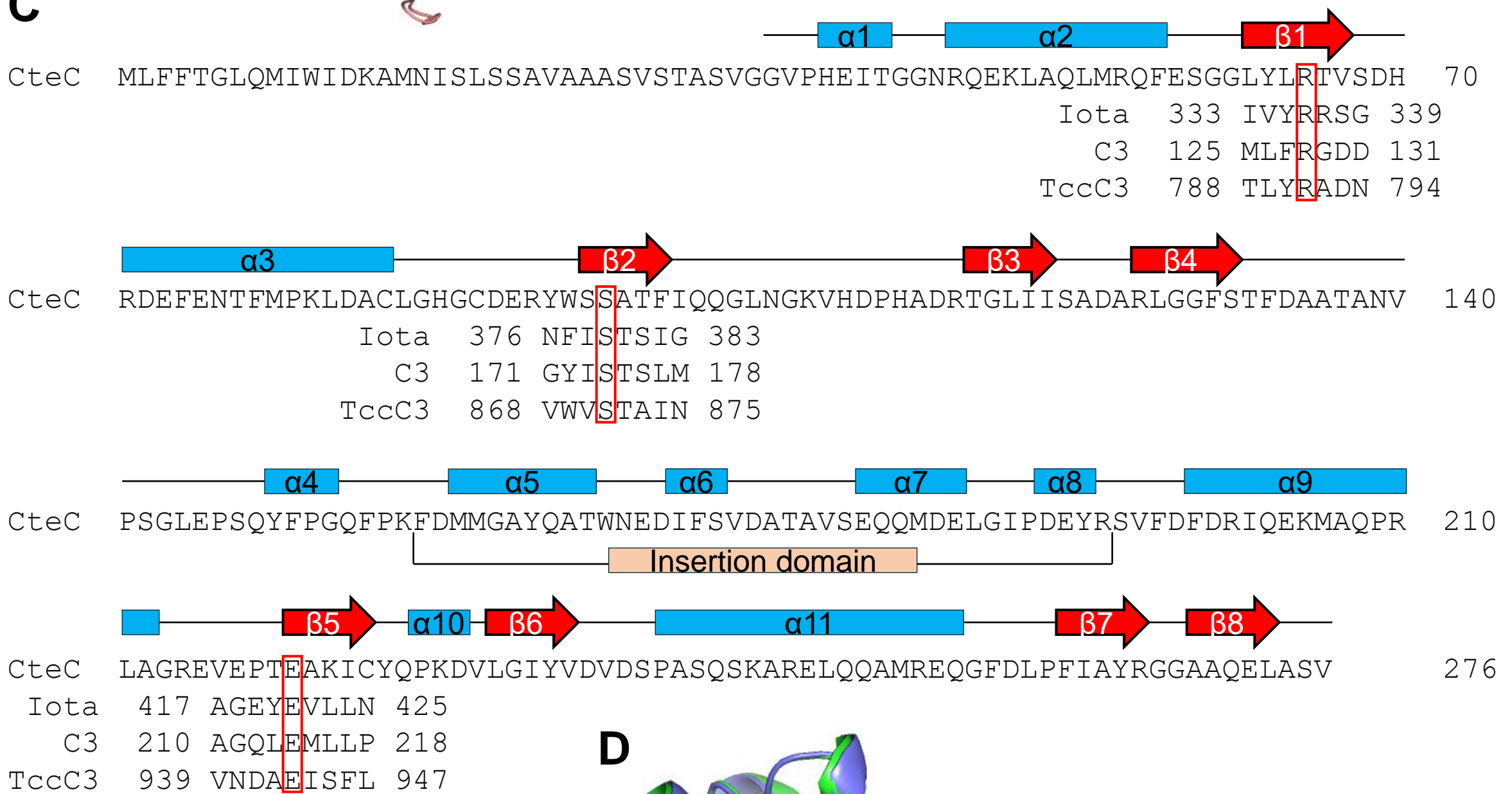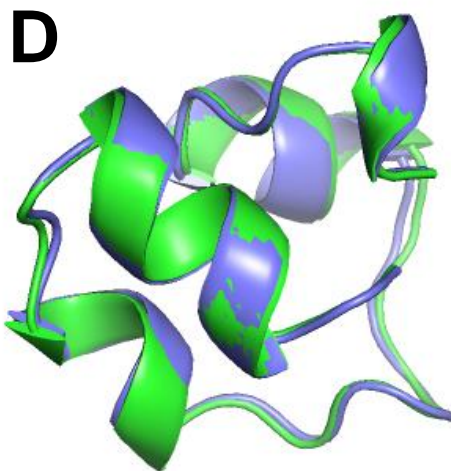

Supplement: Figure S1 [file mmc1.pdf]

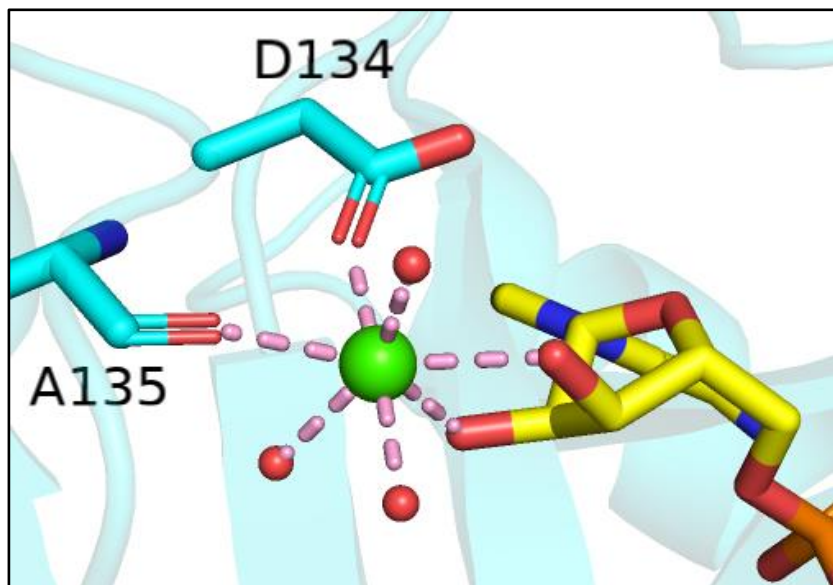

Supplement: Figure S2 [file mmc2.pdf]

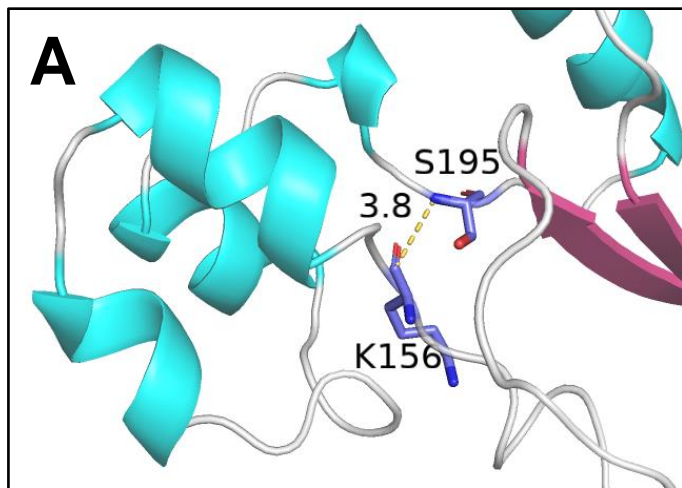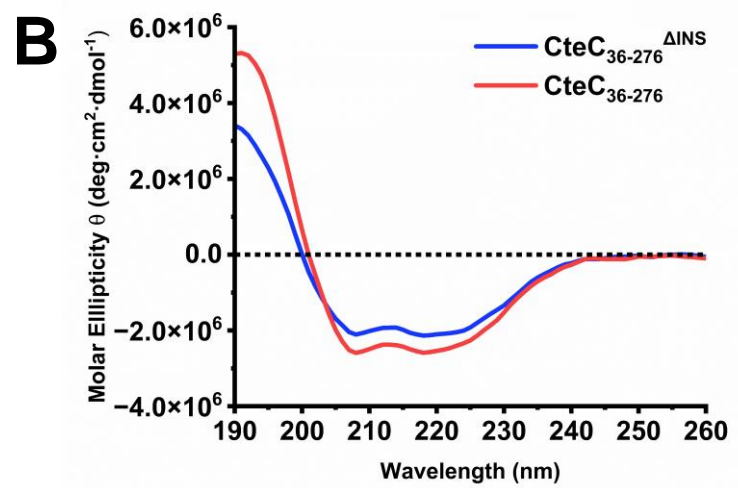

Supplement: Figure S3 [file mmc3.pdf]
