# Supplementary material for: Crystal structure of bacterial ubiquitin ADP-ribosyltransferase CteC reveals a substrate-recruiting insertion
Source: J Biol Chem. 2023 Dec 28;300(2):105604. doi: 10.1016/j.jbc.2023.105604 (PMC10810742; doi:10.1016/j.jbc.2023.105604)
Supplement: Figures and Table Captions [file mmc4.docx]

Supporting information for

Crystal structure of bacterial ubiquitin ADP-ribosyltransferase CteC reveals a substrate-recruiting insertion

Zhengrui Zhang^1^, Hannah M. Rondon-Cordero^1^, Chittaranjan Das^1,^*

1 Department of Chemistry, Purdue University, West Lafayette, Indiana 47907, USA

* Correspondence: Chittaranjan Das, [cdas@purdue.edu](mailto:cdas@purdue.edu)

Figure S1 to S3

Table S1

Figure S1. Structure and sequence comparison of CteC. (A) CteC structure predicted by AlphaFold. (B) Crystal structure of NAD^+^-bound SeMet-CteC_36-276_^ΔINS^ (NAD^+^ removed). (C) Sequence and secondary structures of CteC with insertion domain indicated. Local sequence alignment of R-S-E motifs in CteC, Iota, C3, and TccC3 is shown, with R-S-E motifs highlighted in red boxes. (D) Superposition of CteC insertion domain predicted by AlphaFold (blue) and the one in crystal structure (green).

Figure S2. The Ca^2+^ coordination near the NAD^+^-binding site in CteC. Ca^2+^ is shown as green sphere whereas water molecules are shown as red spheres.

Figure S3. Design and validation of CteC_36-276_^ΔINS^. (A) Positions of K156 and S195 in CteC_36-276_ structure. The distance between K156 carbonyl carbon atom to S195 backbone nitrogen atom is 3.8 Å. (B) Circular dichroism spectrum of CteC_36-276_ and CteC_36-276_^ΔINS^ suggest CteC_36-276_ remain folded upon insertion domain removal.

Table S1. Crystallographic Data collection, processing, and refinement statistics.
