# Supplementary material for: Crystal structure of bacterial ubiquitin ADP-ribosyltransferase CteC reveals a substrate-recruiting insertion
Source: J Biol Chem. 2023 Dec 28;300(2):105604. doi: 10.1016/j.jbc.2023.105604 (PMC10810742; doi:10.1016/j.jbc.2023.105604)
Supplement: Table S1 [file mmc5.docx]

Table S1. Crystallographic Data collection, processing, and refinement statistics.

|  | **NAD^+^-bound SeMet-CteC_36-276_** |
| --- | --- |
| Wavelength (Å) | 0.9794 |
| Resolution range (Å) | 41.47 - 1.871 (1.938 - 1.871) |
| Space group | *P* 2_1_ 2_1_ 2_1_ |
| Unit cell | 57.656 82.937 151.212 90 90 90 |
| Total reflections | 696267 (52314) |
| Unique reflections | 60539 (5877) |
| Multiplicity | 11.5 (8.9) |
| Completeness (%) | 99.81 (98.47) |
| Mean I/sigma(I) | 22.18 (2.57) |
| Wilson B-factor | 20.44 |
| R-merge | 0.302 (2.635) |
| CC1/2 | 0.978 (0.747) |
| Reflections (refinement) | 60532 (5875) |
| Reflections (R-free) | 1999 (194) |
| R-work | 0.1920 (0.2691) |
| R-free | 0.2269 (0.3318) |
| RMS(bonds) | 0.008 |
| RMS(angles) | 1.03 |
| Ramachandran favored (%) | 95.91 |
| Ramachandran allowed (%) | 4.09 |
| Ramachandran outliers (%) | 0.00 |
| Average B-factor | 26.70 |

Values in parentheses refer to the highest-resolution shell of data.
